# Reproducibility and Validity of a Self-Administered Food Safety Assessment Tool on Children and Adolescent’s Risk Perception, Knowledge, and Practices

**DOI:** 10.3390/nu15010213

**Published:** 2023-01-01

**Authors:** Sueny Andrade Batista, Verônica Cortez Ginani, Elke Stedefeldt, Eduardo Yoshio Nakano, Raquel Braz Assunção Botelho

**Affiliations:** 1 Department of Nutrition, School of Health Sciences, University of Brasília (UnB), Campus Darcy Ribeiro, Asa Norte, Brasília 70910-900, DF, Brazil; 2 Department of Preventive Medicine, Federal University of São Paulo (UNIFESP), São Paulo 04023-032, SP, Brazil; 3 Department of Statistics, Institute of Exact Sciences, University of Brasilia (UnB), Campus Darcy Ribeiro, Asa Norte, Brasília 70910-900, DF, Brazil

**Keywords:** reliability, validity, paper-and-pencil questionnaires, online questionnaires, risk perception, children, adolescents

## Abstract

The present study aimed to verify the instrument’s reliability and validity for assessing children and adolescents’ risk perception, knowledge, and food safety practices in the school context. Moreover, it aimed to test the hypothesis that both application methods (paper and pencil (PAPI) and online) are valid. The instrument comprised three questionnaires and followed a strict protocol to combine online and PAPI models, resulting in five application forms. The sample consisted of 439 Brazilian students from 10 to 14 years old (y/o). The results related to reliability and validity indicated that the knowledge questionnaire presented adequate stability and discriminant validity coefficients. The self-reported practices questionnaire obtained acceptable coefficients of stability and internal consistency. Regarding risk perception data, it was observed that students attribute a low probability of Foodborne Diseases (FBD) outbreaks occurrence and low severity of possible symptoms. In addition, students demonstrated insufficient knowledge and inadequate practices on issues related to failures associated with the time and temperature of preparation, storage, and exposure of food, contamination of food, and consumption of unsafe food. In this context, the reproducibility and validity indices need to be interpreted and discussed correctly, and young people in food safety actions are a priority in facing FBD.

## 1. Introduction

World organizations are debating food safety as it plays a primary role in the global population’s health. For example, in the document that defines the global strategies for food security (2022–2030), the World Health Organization (WHO) states that achieving food and nutrition security without food safety is not feasible. Another aspect highlighted in the document was the vulnerability of specific population groups such as children. In these cases, rates of Food Insecurity (FI) and Foodborne Diseases (FBD) outbreaks always stand out compared to other groups [1,2].

Prioritizing actions that protect children and adolescents can be crucial to minimizing the effects of FI exacerbated by the COVID-19 pandemic. In Brazil, with the COVID-19 pandemic, extreme nutritional status emerged, which must be discussed from different perspectives [3]. In this context, during 2021 and 2022, the VIGISAN survey showed that increasing degrees of moderate and severe FI in households were proportional to the presence of residents under 18 y/o. In addition, water insecurity, strongly associated with FI, was also identified in 12% of households [4]. This is an alarming fact as water is as important as food; moreover, it is involved in FBD outbreaks since it is used in cleaning and food production [5].

Research should be encouraged to help formulate strategies to mitigate food insecurity among children and adolescents, such as ensuring food safety. More comprehensively, studies have proposed investigating the actors involved in ensuring food safety, especially food handlers and adult consumers [6,7,8]. The results guide actions to control FBD outbreaks, an important public health problem worldwide. Researchers usually use assessment instruments to evaluate knowledge and practices, and the results show a gap between them [6,9,10]. Therefore, other factors, such as the cognitives, are also of scientific interest once they generate attitudinal ambivalence toward food handling. In this sense, assessment instruments that include risk perception contribute to more robust studies [11,12,13,14,15,16,17].

The risk perception indicates the susceptibility to a harmful event and its severity. For this reason, it is essential to motivate changes in behavior [18]. Zanetta et al. highlighted the importance of understanding the consequences and severity that shape risk perception. The study’s results demonstrated that consumers underestimate the risk for FBD when eating out and at home. Notably, this group of diseases’ largest share of outbreaks occurs in the domestic environment [19]. In another study, Zanin et al. evaluated the strengths and weaknesses of an institutional food service’s prevailing food safety culture. Authors identified the relevance of prioritizing the topic of “risk perception” in developing educational actions, given the low scores [13].

Therefore, understanding individuals’ risk perception is essential for successful communication, with more direct protection messages to consumers, within a diagnostic perspective for effective interventions that consider the individual–environment interaction [20].

Information regarding risk perception of food safety among young people is scarce. Despite the progress of evaluations on the subject, there is a significant gap in the data. Moreover, previous studies with children, students, and young people suggest a lack of knowledge, interest, and perceived susceptibility to FBD [21,22,23,24,25]. Investigating this group’s risk perception and optimistic bias could help clarify causal factors. Furthermore, promoting education to facilitate a conscious future population must consider how people think and respond to risks [26,27].

Additionally, efforts should focus on acquiring knowledge, experience, confidence, and self-efficacy [28]. One way to achieve this goal is to design studies that initially comprise the target population. If there is no such understanding, the formulated policies may be ineffective. Children and adolescents play an essential role in the family’s eating behavior, including being able to participate in food preparation. Another important aspect is the cognitive moment that the group experiences. Investments in educational actions aimed at this audience can reflect on their future if adequately planned and executed [21,29,30,31,32].

Before planning the action, there must be a diagnosis of the audience. Then, some steps must be fulfilled to develop an instrument capable of consolidating all the items for an adequate evaluation. According to Pasquali, the evaluation instrument must first be submitted to a construct evaluation. Then, its reliability and validity must be guaranteed with the development of empirical steps and statistics [33].

Thus, the present study aims to verify the instrument’s reliability and validity for assessing children and adolescents’ risk perception, knowledge, and food safety practices in the school context developed by Batista et al. [34]. Furthermore, the hypothesis is that both application methods (paper and pencil (PAPI) and online) are valid. In this way, it will later be possible to have a food safety diagnosis capable of directing actions to the young audience within their sociocultural context and structured considering the psychological field.

## 2. Materials and Methods

A cross-sectional and quantitative study was conducted between August/2021 and August/2022 in the Federal District-Brazil and approved by the Ethics Committee of the Faculty of Health Sciences of the University of Brasília-CEP/FS UnB (CAAE No 02033218.0.0000.0030). The sample consisted of 439 students aged 10 to 14 y/o from public (*n* = 4) and private (*n* = 1) schools, with the participation of a public school located in a rural area. The schools were conveniently selected, and the students’ participation was conditioned to the presentation of the consent terms signed by parents/legal guardians and free and informed consent signed by the students.

This research continued the study by Batista et al. [34], who developed the theoretical steps proposed by Pasquali [35] to create a research instrument. The instrument is a tool composed of three questionnaires that assess risk perception, knowledge, and food safety practices (Supplementary Material Figure S1). It presented the guarantees of content validity, understanding, and apparent validity (credibility). The “risk perception” questionnaire considers the school environment, given the possibility of simultaneously evaluating the student and the reality observed in the school [34].

Batista et al. could not complete the empirical and statistical steps due to the advent of the SARS-CoV-2 pandemic. However, these steps are necessary to obtain accurate, valid, and interpretable data. Furthermore, it is essential to conclude them due to the scarcity of literature that addresses the perception of food safety risk in the target audience, as well as for being the only instrument in Portuguese that proposes to evaluate factors and constructs related to the theme with children and adolescents [34].

Therefore, two steps were necessary for this study: (i) empirical procedures, with the application of the instrument considering the online and physical environments; and (ii) statistical procedures, assessing instrument reliability and validity.

The first step included the sample definition, test instructions, and instrument administration. Then, five applications were performed according to the order of the instrument presentation: (i) online then PAPI (*n* = 24), (ii) PAPI then online (*n* = 21), (iii) Online then online (*n* = 22), (iv) PAPI then PAPI (*n* = 31), and (v) a single application—online or PAPI (*n* = 341). All steps were developed in the school and the virtual environment, with the support of the Google Form platform.

The second step was necessary to conduct a more in-depth assessment of the instrument’s measurement properties and guarantee the quality of the results [35]. For this, stability and internal consistency criteria were adopted to determine the instrument’s reliability. Validity was assessed using discriminant validity.

The first four application methods were performed in the first step of the study to evaluate the stability coefficient using the test-retest method. Thus, it was possible to verify the degree to which similar results were obtained at two different times [36]. Furthermore, the fifth application was performed to verify the internal consistency of the knowledge questionnaires and “self-reported practices”—another reliability measure. Therefore, the sample to determine this measure consisted of the first application carried out in four phases and the final fifth stage (*n* = 439).

To obtain the discriminant validity of the knowledge questionnaire, we invited dietitians and undergraduate nutrition students from the last semester to compose the group of nutrition specialists (*n* = 41). The target audience comprised the lay group, using the consistency analysis sample (*n* = 439). The procedure must demonstrate that the instrument items measure the desired content domain [36]. To this end, a comparison was made between the two groups.

**Figure 1:**
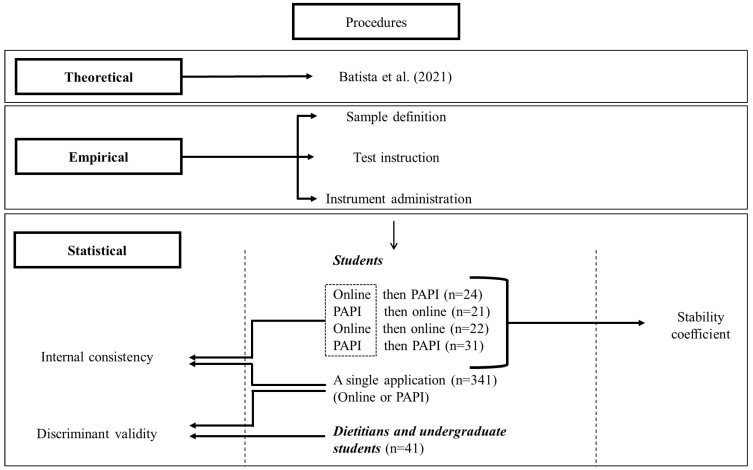
Steps for instrument validation [34].

The developed steps are summarized in Figure 1.

The “risk perception” construct coefficients were not determined, as it is considered that the person assesses the risk in the face of the current situation. The perception is so subjective that it can change quickly, impacting the reproducibility of the results. The risks in the present are assessed immediately, so we must consider the impact bias/affect heuristic [37].

Judging risk is directly related to people’s feelings about risks, and their acceptance [28,38,39,40]. It considers social constructions, cognitive factors, beliefs, and motivations [41,42,43]. Other characteristics, such as individual differences and demographic factors, can also influence comparative perceptions of the magnitude of risks [44]. Therefore, assessing the reproducibility of a subjective, multidimensional construct anchored in several factors becomes challenging.

### Statistical Analysis

Data were analyzed using the statistical program IBM SPSS version 22.0 and the Microsoft Excel Program (2007). The Kuder–Richardson Formula 20 (KR-20) was used to determine the stability measure of the “knowledge” variable; values KR-20 ≥ 0.7 indicate good stability [45]. This was used because it is the most adequate for dichotomic variables. For the “practices” questionnaire, the Intraclass Correlation Coefficient (ICC) was used. According to Cicchetti’s classification, the following reference values were used: ICC ≥ 0.75 were considered excellent, 0.60 ≤ ICC < 0.75 good, 0.40 ≤ ICC < 0.60 fair, and ICC < 0.40 poor [46]. The internal consistency of the “knowledge” and “self-reported practices” questionnaires was determined by Cronbach’s alpha, whereby the scores were interpreted as such: α ≥ 0.9 was considered excellent, 0.7 ≤ α < 0.9 good, 0.6 ≤ α < 0.7 acceptable, 0.5 ≤ α < 0.6 poor and α < 0.5 unacceptable [47,48,49]. Finally, discriminant validity was assessed using unpaired *t*-tests.

Descriptive analyses of mean, standard deviation, percentage distribution of socioeconomic data, risk perception, knowledge, and food safety practices were performed.

## 3. Results

**Figure 2:**
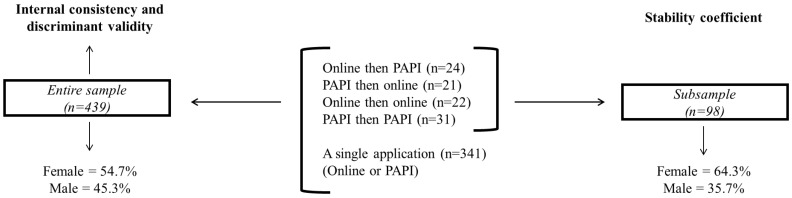
Samples and their respective statistical analyses.

The study included 439 students aged 10 to 14 y/o (female = 54.7%; male = 45.3%) with a mean age of 12.1 years (±1.36), enrolled in public (49.2%) and private (50.8%) schools. The entire sample (*n* = 439) was used to analyze internal consistency and discriminant validity. A subsample (*n* = 98) was used to verify the stability coefficient. For this reason, the instrument was applied at an interval of 11 days. The second sample comprised 64.3% female and 35.7% male, with a mean age of 12.1 years (±1.8). The samples and their respective statistical analyses are described in Figure 2.

The results of the stability and internal consistency coefficients are presented in Table 1.

In the test of discriminant validity for the knowledge questionnaire, on average, the group of experts (9.76, ±0.99) presented higher knowledge than the lay group (6.55, ±1.73). This difference (−3.21) was significant with *p* = 0.000.

The application of the instrument (*n* = 439) resulted in data on risk perception (Table 2 and Table 3), knowledge (Table 4), and self-reported practices (Table 5) of food safety.

In the first part of the instrument, the risk perception questions consider its two dimensions—probability and severity. As for the first, it can be seen in Table 2 that most students identified as “very low” or low the chances of themselves (72.7%) or a colleague (61%) becoming sick due to having eaten a snack served at the school where they study. As for severity, most students believe that they would present symptoms with no or low severity if they became ill after eating food at school.

## 4. Discussion

### 4.1. Reliability and Validity Coefficients

Studies that investigate the invariance of measures in different ways of applying an instrument use online and face-to-face methods [50,51,52,53]. The multiple applications make it possible to observe if there is a consistent performance in different situations, according to values established in the literature. However, it becomes crucial to consider the value’s meaning within the research context [54].

Singh and Sagar argued that it is necessary to compare results obtained traditionally and validation studies to establish psychometric properties to reach conclusions from online methods. For this, using the same study sample or sampling frame is recommended. In this way, it will be possible to show whether or not there are significant differences between the methods [55].

The “knowledge” and “practices” constructs obtained good and excellent (KR-20 and ICC > 0.7) levels of stability [46], except for the “PAPI then online” application of the “knowledge” construct, which showed a low level of stability (KR-20 = 0.452). Since this application was used as a complementary form of analysis, it is considered that adequate stability coefficients were obtained for the construct’s “knowledge” and “practices”. Thus, it is observed that in different environments the results are similar.

Other studies that did not necessarily use the same coefficients to assess the stability of instruments but tested the same forms of application also found similar results between different models. Different authors have supported the comparability of the reliability of online and PAPI questionnaires [56,57,58].

In their research, Van de Looij-Jansen and de Wilde evaluated the differences in the responses of mental health and behavior indicators by an ANCOVA test. No significant differences were found between the responses online and PAPI methods. However, it is essential to emphasize that, in the mentioned study, each method was applied to a different group of subjects, differently from our research [59]. Likewise, Gorrasi et al., applying the two methods to different groups, evaluated whether the two approaches could influence the results of the three questionnaires. The results revealed no significant differences in the Orthorexia Nervosa and Muscle Dysmorphia scales [60].

In contrast, in agreement with this study, Ward et al. examined differences between two data collection techniques with the same group of individuals. Among the evaluated scales, three showed no significant differences in the scores of the two approaches. Therefore, the literature presents data that corroborates the current study’s findings [61].

Regarding the “knowledge” construct, according to Cronbach’s alpha coefficient, a value of 0.370 was obtained, indicating low internal consistency. When considering construct validity, which assesses the extent to which the set of items represents the construct to be measured [36], it is noted that the knowledge questionnaire encompasses different types of knowledge in food safety, such as (i) hand hygiene, (ii) cross-contamination, (iii) adequate food storage, (iv) consumption of raw or undercooked foods, v) exposure time of food at room temperature, (vi) unsafe foods, (vii) the presence of vectors and urban pests, (viii) cleaning food consumed raw, and (ix) pesticides, thus affecting the index. Therefore, the heterogeneity of knowledge regarding food safety can lead to low Cronbach’s alpha coefficients.

Corroborating the previous finding, other studies that obtained internal consistency indexes below the recommended (0.45 and 0.55) consider the findings adequate given the limited number of items, a wide range of knowledge, and different addressed concepts [62,63]. For example, in a study of cultural adaptation of an instrument to assess the quality of life of Brazilian adolescents (12 to 16 y/o), Teixeira et al. obtained internal consistency values (Cronbach’s alpha) that ranged from 0.12 to 0.73. The authors pointed out that possible reasons for the low values include the presence of subjective constructs and heterogeneous content, which is in line with the present study [64].

As for the coefficient of the practice’s questionnaire (Cobrach’s alpha = 0.612), the value is considered acceptable [48,49]. Nunnally suggests that in the initial stages of the research, values from 0.7 to 0.5 are sufficient [65]. Van Griethuijsen et al. consider an acceptable reliability level when values range from 0.6 to 0.7 [66]. Thus, this study’s value is adequate. The exclusion of items with low internal consistency in the knowledge and practice questionnaires was tested to verify the increase in reliability. However, no acceptable values were found with the exclusion of items. 

Finally, concerning the validity of the knowledge questionnaire, the results suggest that the items presented high discriminant validity. Thus, they are adequate in assessing knowledge about food safety. Guadagnin et al. use this assessment to validate a questionnaire assessing knowledge in nutrition and its usefulness in assessing changes in knowledge among participants in a Nutrition Education Program applied in the workplace. In this study, the expert group scores were significantly higher than those of the lay group in all domains [67].

### 4.2. Application of the Instrument

In the present study, it was not possible to assess the risk perception since the sanitary risk of the schools was not verified. However, it is inferred that low probability and severity results may be due to the students’ feelings about the school environment [28,38]. For example, the study by de Andrade et al. evaluated consumers’ risk perception and optimistic bias in commercial restaurants. The results indicated their optimism about the restaurant where they were interviewed, attributing similar risk to food prepared at home and in the studied restaurants. The authors pointed out that consumers may have incorporated a feeling of affection and identity into the place, corresponding to having their meals at home [6].

Regarding the perception of lethality, it is observed that 34.7% of the students consider a person’s chance of dying when consuming contaminated food as very low or low. A study developed by Ovca et al. carried out with individuals between 10 and 12 years old pointed out that most participants agreed that food poisoning could be fatal, indicating a high perception of lethality, in contrast to our results. The study by Ovca et al. was developed in Slovenia, and its result can be attributed to the country’s degree of development [68]. According to the Organisation for Economic Co-operation and Development (OECD) and the Programme for International Student Assessment (PISA), this country has one of the highest socioeconomic, social, and cultural statuses [69].

In the knowledge questionnaire, the pattern of the results showed a mean correctness of 60%, a minimum average of 9.1%, and a maximum of 100%. Other studies also found median results ranging from 65.8% to 72% [21,31,68]. The study carried out in Australia with students aged 11 and 18 presented an average of 42% [23].

The results presented above are in line with what is discussed by Syeda et al. [26]. The authors showed that previous studies with children, young people, and university students in Europe, Australia, Canada, and the United States suggest a lack of knowledge, interest, and perception of susceptibility to FBD, pointing up the importance of working with these individuals.

It is observed from the knowledge questionnaire that 43.7% of students are unaware of the consequences of violating the time versus temperature binomial and that 29.4% carry out inappropriate practices related to the theme. In the study by Ovca, Jevšnik and Raspor, only 48.8% of respondents reported storing leftover food in the refrigerator [68]. Similar results were found with food handlers, with inadequate practices related to this binomial during food exposure and insufficient knowledge about temperature control [6,70]. It should be noted that the violation resulting from the lack of control of the time versus temperature binomial in food production is considered the leading global risk factor for FBD [71]. Therefore, understanding the subject is essential.

It should be noted that the issue of unsafe food needs more attention. A significant portion of the students missed questions about the consumption of raw eggs or eggs with soft yolk (57.4%), consumption of fruits without prior cleaning (40.8%), and consumption of fruits and vegetables with pesticides (43.1%). As for practices, 29.9% of students consume raw eggs or eggs with soft yolks with some frequency. Students demonstrated knowledge about the implications of consuming spoiled foods (95.9%). However, 59.2% believe that removing the moldy part of the bread reduces or eliminates the risk of getting sick, and 19.8% reported consuming this food after removing the spoiled part.

It is also noted that 72.9% believe that unsafe foods always have undesirable characteristics, and 48.1% consume foods with expiry dates as long as they look good. In the studies by Batista et al. and Ovca et al., the students considered the appearance and smell of food as reliability indicators for its consumption [34,72]. Cheng et al. pointed out that some students thought that expired foods could be consumed after boiling or heating or while they looked good [73]. Efforts should be focused on this theme, as contamination by pathogenic microorganisms does not change the characteristics of food.

It is expected that the studied age group has cognitive prerequisites that allow them to consider that appearance differs from reality and that there are invisible particles (microorganisms) since such cognitive skills are developed in the infants [74,75,76,77]. This result may be related to the possible difficulty of human beings attributing risk to what cannot be seen, as in the case of microorganisms. However, it is necessary to assess more broadly how these skills are being worked with students. 

Another relevant result (67% of the students) indicates the lack of knowledge of the correct practice of how to sanitize raw vegetables. Other studies report insufficient knowledge about vegetable hygiene [34,78]. The results of Burke and Dworkin indicate that 86% of the students believed that it was sufficient to rinse with water a salad contaminated with raw chicken juice [78].

Notably, these foods are related to an increasing number of FBD outbreaks worldwide, with a high occurrence of Salmonella [79,80]. A study developed in China suggested that consuming raw vegetables represents a potential source of human infection by salmonellosis [81]. Outbreaks were related to the consumption of melon [82], watermelon [83], apple, tomato, celery, lettuce, radish, and orange [84,85]. The correct hygiene procedure effectively contributes to mitigating outbreaks related to these foods.

The third part of the instrument evaluated students’ self-reported food safety practices. As for hand hygiene, only 42.4% of the students reported consistently washing them before a meal. Therefore, it is necessary to reflect and investigate whether the public knows the importance of carrying out this practice. Simply recognizing that the process must include soap/detergent may not be enough to support educational actions. Contrary to these results, Osei-Tutu et al. found that many students (70%) indicated hand hygiene before meals. In this study, handwashing education is part of the School Health Education Program in conjunction with the WASH Program at the school in the studied district [10].

The data referring to question “nine” point to students’ expressive participation in preparing food or meals in the home environment (96.4%). Other studies presented comparable data, such as by Ovca et al., Haapala et al., and Byrd-Bredbenner et al., in which 84.2%, 92%, and 95% of individuals are included in food preparation at home, respectively [21,28,68]. In Ovca et al., 21.5% of children and adolescents dealt with potentially dangerous items such as meat, fish, and green leaves [68]. Therefore, investigating which types of food are often involved in their daily handling practices is critical.

The study by Thakadu et al. evaluated the hygiene education of students aged between eight and 16, with the participation of teachers. These professionals highlighted challenges for hygiene education, such as time constraints to adequately address the topic, inadequate prior knowledge about health education, and lack of health material to guide them [86]. Given the observed results and reflecting on the provision of knowledge in schools, the methodological aspects in this environment must be considered since the pedagogical body is responsible for ensuring food safety, as it is a shared responsibility [87]. Therefore, the importance of training the teaching staff is reinforced.

When considering adult handlers and consumers, unsafe handling practices are often perceived, despite their taking place with an acceptable level of knowledge [6,9,16,88]. It is suggested that if the theme is worked in the appropriate cognitive phase, considering psychological aspects such as risk perception, the behaviors would be well established, with sufficient knowledge and good manipulation practices.

## 5. Conclusions

This study completed the validation steps of the instrument developed by Batista et al., thus presenting guarantees of reliability (stability and internal consistency) and validity (discriminant validity) [34]. The internal consistency result of the knowledge questionnaire does not invalidate the quality of the instrument, given the nature of the studied variable. It is necessary to reflect on the nature of the investigated constructs and the meaning of the reliability parameters in the real context of the research. The general guidelines established in the literature do not consider the specificities and subjectivities included in the studies.

The study confirms the hypothesis that the different application methods (online and PAPI) are valid. The potential use of the online version stands out, as it allows for faster data return, cost reduction, and convenience bias. This practical value is being evidenced in the ongoing COVID-19 pandemic.

Considering the data presented and studies aimed at the adult audience, it is inferred that if the theme is worked in the appropriate cognitive phase, considering psychological aspects such as risk perception, the behaviors would be well established, with sufficient knowledge and good manipulation practices.

Using a valid and reliable instrument, well-formulated strategies can be carried out considering the young audience, a fundamental link in the food chain to mitigate Foodborne Diseases. In this way, fighting food insecurity arising from the lack of food safety is essential. Finally, the limits of this study are represented by the nature of the investigated constructs, the lack of critical literature that allows the results to be reflected broadly, and the social desirability when responding.

## Figures and Tables

**Table 1:** Reliability and validity coefficients of the instrument for assessing knowledge and practices related to food safety.

	Stability: Test-Retest				Internal Consistency (Alpha Cronbach)
Questionnaires	O then PAPI	PAPI then O	O then O	PAPI then PAPI	O or PAPI
	(*n* = 24)	(*n* = 21)	(*n* = 22)	(*n* = 31)	(*n* = 439)
Knowledge ^a^	0.814	0.452	0.770	0.903	0.395
Self-reported practices ^b^	0.722	0.752	0.833	0.888	0.615

^a^ Kuder–Richardson; ^b^ Intraclass correlation coefficient; O: online application; PAPI: paper-and-pencil application.

**Table 2:** Relative and absolute frequencies of items refer to the “probability” dimension (A1 and A2) and the perception of lethality (A3) of the risk perception questionnaire.

Items	Very Low	Low	Average	High	Very High
A1. What is the chance that YOU will get sick from eating food served at your school?	166 (37.8%)	153 (34.9%)	95 (21.6%)	19 (4.3%)	6 (1.4%)
A2. What is the chance that a COLLEAGUE who studies with you will get sick from having eaten the same food served at your school?	94 (21.4%)	174 (39.6%)	139 (31.7%)	26 (5.9%)	6 (1.4%)
A3. What is the chance that a person will die from eating contaminated food?	71 (16.2%)	81 (18.5%)	125 (28.5%)	110 (25.1%)	51 (11.6%)

**Table 3:** Relative and absolute frequencies of responses to the “severity” dimension (A.1 and A2.1) of the risk perception questionnaire.

Items	No Severity	Low Severity	Medium Severity	High Severity
A1.1 If YOU get sick from eating food served at the school you study, how serious could it be?	72 (16.4%)	217 (49.5%)	141 (32.2%)	8 (1.8%)
A2.1 If YOUR COLLEAGUE gets sick from eating food served at the school you study, how serious could it be?	49 (11.2%)	220 (50.2%)	152 (34.7%)	17 (3.9%)

**Table 4:** Relative and absolute frequencies of responses to the knowledge questionnaire.

Items	Wrong	Correct
B1. Do you always need to use soap/soap/detergent to wash your hands correctly?	9 (2.1%)	430 (97.9%)
B2. Is using a paper towel to clean a dirty board of raw meat enough to be able to use this board to cut bread?	67 (15.3%)	372(84.7%)
B3. Should raw meats be kept in the refrigerator on shelves below ready-to-eat foods?	341 (77.7%)	98(22.3%)
B4. Eating a raw egg or soft yolk can make you sick?	252 (57.4%)	187 (42.6%)
B5. Eating food that was out of the fridge for a long time after it was done can make you sick?	192 (43.7%)	247 (56.3%)
B6. Eating foods with a bad smell, bad taste, different texture than usual or moldy, can make you sick?	18 (4.1%)	421 (95.9%)
B7. Removing the moldy part of bread before eating reduces or eliminates the chance of you becoming ill?	179 (40.8%)	260 (59.2%)
B8. Eating food made in a kitchen that contains flies and other insects can make you sick?	89 (20.3%)	350 (79.7%)
B9. To eat raw fruits and vegetables, do you need to wash them using bleach?	294 (67%)	145 (33%)
B10. Can eat fruits and vegetables that have been grown with pesticides make you sick?	189 (43.1%)	250 (56.9%)
B11. Does unsafe food to eat always smell foul, look strange, and have a different texture?	320 (72.9%)	119 (27.1%)

**Table 5:** Relative and absolute frequencies of responses to the practice’s questionnaire.

	Never	Rarely	Sometimes	Often	Always
(C1) Do you wash your hands with soap and water/soap/detergent before eating	6 (1.4%)	15 (3.4%)	112 (25.5%)	120 (27.3%)	186 (42.4%)
(C2) When you open a milk carton, do you leave it out of the fridge for more than an hour?	310 (70.6%)	87 (19.8%)	32 (7.3%)	5 (1.1%)	5 (1.1%)
(C3) Do you store food in the refrigerator in closed packages or containers with a lid?	14 (3.2%)	16 (3.6%)	82 (18.7%)	118 (26.9%)	209 (47.6%)
(C4) Before eating the food, do you look at the expiration date on the packaging?	20 (4.6%)	43 (9.8%)	89 (20.3%)	93 (21.2%)	194 (44.2%)
(C5) Do you eat expired foods that have a good smell, normal appearance, and texture?	228 (51.9%)	111 (25.3%)	72 (16.4%)	14 (3.2%)	14 (3.2%)
(C6) Do you eat raw or soft yolk eggs?	308 (70.2%)	57 (13%)	36 (8.2%)	27 (6.2%)	11 (2.5%)
(C7) Do you eat bread after removing a moldy part?	352 (80.2%)	41 (9.3%)	28 (6.4%)	9 (2.1%)	9 (2.1%)
(C8) Do you eat fruits without washing them?	260 (59.2%)	89 (20.3%)	65 (14.8%)	17 (3.9%)	8 (1.8%)
(C9) Do you help in preparing food or food at home?	16 (3.6%)	61 (13.9%)	195 (40.4%)	109 (24.8%)	58 (13.2%)
(C10) Do you wash your hands with soap and water/soap/detergent before preparing or helping to prepare meals or food?	3 (0.7%)	13 (3.1%)	27 (6.4%)	56 (13.2%)	324 (76.6%)
(C11) Do you check if the benches or tables you are going to use are clean before preparing meals or food?	11 (2.6%)	22 (5.2%)	55 (13.0%)	95 (22.5%)	240 (56.7%)

## Data Availability

Not applicable.

## Supplementary Materials

The following supporting information can be downloaded at: https://www.mdpi.com/article/10.3390/nu15010213/s1, Figure S1: The instrument developed by Batista et al. (2021) used in the research.
