# Supplementary material for: Reproducibility and Validity of a Self-Administered Food Safety Assessment Tool on Children and Adolescent’s Risk Perception, Knowledge, and Practices
Source: Nutrients. 2023 Jan 1;15(1):213. doi: 10.3390/nu15010213 (PMC9823607; doi:10.3390/nu15010213)
Supplement: Supplementary file 1 [file nutrients-15-00213-s001.zip › nutrients-2080794-supplementary.pdf]

School____ Code________ (filled in by the researcher)

Series ________ Age: ________ years old Area where you live: (   ) Urban (   ) Rural

Gender: (   ) Female (   ) Male Date: ____ / ____ / 202___

Make an X ONLY A RECTANGLE that matches your answer.

ATTENTION: GETTING SICK = having stomach pain and/or diarrhea and/or vomiting and/or headache and/or fever because you ate contaminated food.

1. What is the chance that YOU will get sick from eating food served at the school you study?

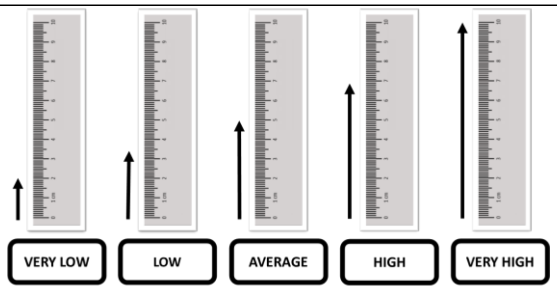

1.1 If YOU get sick from eating food served at the school you study, how serious could it be?

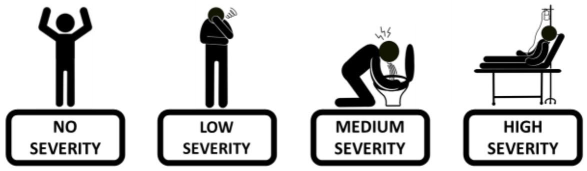

2. What is the chance that a COLLEAGUE who studies with you will get sick from having eaten the same food served at the school you study?

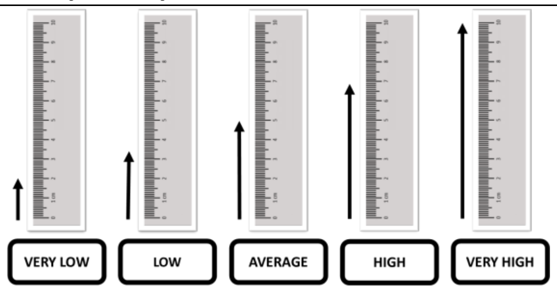

2.1 If YOUR COLLEAGUE get sick from eating food served at the school you study, how serious could it be?

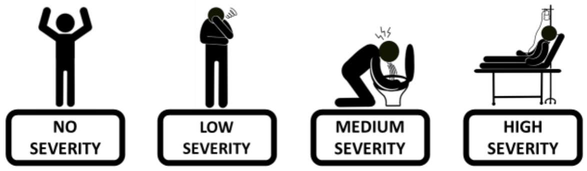

3. What is the chance that a person will die from eating contaminated food?

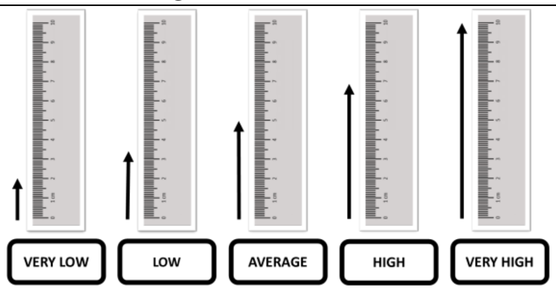

Examples of how food can be contaminated:

- The presence of animal feces in the area where fruits and vegetables are planted;
- The use of pesticides to produce and grow food (fruits, vegetables, cereals such as corn, etc.);
- Due to people's lack of hygiene when preparing food.

Code ________ (filled in by the researcher)

Make an X ONLY A RECTANGLE that matches your answer.

ATTENTION: GETTING SICK = having stomach pain and/or diarrhea and/or vomiting and/or headache and/or fever because you ate contaminated food.

1) Do you always need to use soap/soap/detergent to wash your hands correctly?

YES | NOT | DO NOT KNOW

2) Is using a paper towel to clean a dirty board of raw meat enough to be able to use this board to cut bread?

Enough: when you don't need to do anything else.

YES | NOT | DO NOT KNOW

3) Should raw meats be kept in the refrigerator on shelves below ready-to-eat foods?

Examples of ready-to-eat foods: cake, cooked rice, baked beans.

YES | NOT | DO NOT KNOW

4) Eating a raw egg or soft yolk can make you sick?

YES | NOT | DO NOT KNOW

5) Eating food that was out of the fridge for a long time after it was done can make you sick?

Ex: Food prepared for lunch that stayed until dinner time on the stove.

YES | NOT | DO NOT KNOW

6) Eating foods with a bad smell, bad taste, different texture than usual or moldy, can make you sick?

YES | NOT | DO NOT KNOW

7) Removing the moldy part of bread before eating reduces or eliminates the chance of you becoming ill?

YES | NOT | DO NOT KNOW

8) Eating food made in a kitchen that contains flies and other insects can make you sick?

YES | NOT | DO NOT KNOW

9) To eat raw fruits and vegetables, do you need to wash them using bleach?

YES | NOT | DO NOT KNOW

10) Can eat fruits and vegetables that have been grown with pesticides make you sick?

YES | NOT | DO NOT KNOW

11) Does unsafe food to eat always smell foul, look strange, and have a different texture?

Unsafe food: food that can make you sick.

YES | NOT | DO NOT KNOW

Code ________ (filled in by the researcher)

Make an X ONLY A RECTANGLE that matches your answer.

Never = never did this action<br>Rarely = does this action from time to time<br>Sometimes = do this action sometimes<br>Often = do this action many times<br>Always = this action is part of your daily life

1) Do you wash your hands with soap and water/soap/ detergent before eating?

NEVER | RARELY | SOMETIMES | OFTEN | ALWAYS

2) When you open a milk carton, do you leave it out of the fridge for more than an hour?

NEVER | RARELY | SOMETIMES | OFTEN | ALWAYS

3) Do you store food in the refrigerator in closed packages or containers with a lid?

NEVER | RARELY | SOMETIMES | OFTEN | ALWAYS

4) Before eating the food, do you look at the expiration date on the packaging?

NEVER | RARELY | SOMETIMES | OFTEN | ALWAYS

5) Do you eat expired foods that have a good smell, normal appearance, and texture?

NEVER | RARELY | SOMETIMES | OFTEN | ALWAYS

6) Do you eat raw or soft yolk eggs?

NEVER | RARELY | SOMETIMES | OFTEN | ALWAYS

7) Do you eat bread after removing a moldy part?

NEVER | RARELY | SOMETIMES | OFTEN | ALWAYS

Code ________ (filled in by the researcher)

8) Do you eat fruits without washing them?

Examples of fruit: apple, grape, orange.

NEVER RARELY SOMETIMES OFTEN ALWAYS

9) Do you help in preparing food or food at home?

NEVER RARELY SOMETIMES OFTEN ALWAYS

IF YOU SCORE IN THE PREVIOUS QUESTION, THE OPTIONS RARELY, SOMETIMES, OFTEN OR ALWAYS OR ALWAYS, ANSWER THE NEXT QUESTIONS:

10) Do you wash your hands with soap and water/soap/detergent before preparing or helping to prepare meals or food?

NEVER RARELY SOMETIMES OFTEN ALWAYS

11) Do you check if the benches or tables you are going to use are clean before preparing meals or food?

NEVER RARELY SOMETIMES OFTEN ALWAYS

12) What did you think of this questionnaire? Would you have any comments to improve it?

____

____

____

Thank you! 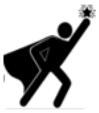

Figure S1. The instrument developed by Batista et al. (2021) used in the research.
